# High-Resolution Mutational Profiling Suggests the Genetic Validity of Glioblastoma Patient-Derived Pre-Clinical Models

**DOI:** 10.1371/journal.pone.0056185

**Published:** 2013-02-18

**Authors:** Shawn E. Yost, Sandra Pastorino, Sophie Rozenzhak, Erin N. Smith, Ying S. Chao, Pengfei Jiang, Santosh Kesari, Kelly A. Frazer, Olivier Harismendy

**Affiliations:** 1 Bioinformatics and Systems Biology Graduate Program, University of California San Diego, La Jolla, California, United States of America; 2 Division of Genome Information Sciences, Department of Pediatrics and Rady Children’s Hospital, University of California San Diego, La Jolla, California, United States of America; 3 Department of Neurosciences, University of California San Diego, La Jolla, California, United States of America; 4 Translational Neuro-oncology Laboratories, University of California San Diego, La Jolla, California, United States of America; 5 Moores UCSD Cancer Center, University of California San Diego, La Jolla, California, United States of America; 6 Clinical and Translational Research Institute, University of California San Diego, La Jolla, California, United States of America; 7 Institute for Genomic Medicine, University of California San Diego, La Jolla, California, United States of America; Ospedale Pediatrico Bambino Gesu’, Italy

## Abstract

Recent advances in the ability to efficiently characterize tumor genomes is enabling targeted drug development, which requires rigorous biomarker-based patient selection to increase effectiveness. Consequently, representative DNA biomarkers become equally important in pre-clinical studies. However, it is still unclear how well these markers are maintained between the primary tumor and the patient-derived tumor models. Here, we report the comprehensive identification of somatic coding mutations and copy number aberrations in four glioblastoma (GBM) primary tumors and their matched pre-clinical models: serum-free neurospheres, adherent cell cultures, and mouse xenografts. We developed innovative methods to improve the data quality and allow a strict comparison of matched tumor samples. Our analysis identifies known GBM mutations altering *PTEN* and *TP53* genes, and new actionable mutations such as the loss of *PIK3R1*, and reveals clear patient-to-patient differences. In contrast, for each patient, we do not observe any significant remodeling of the mutational profile between primary to model tumors and the few discrepancies can be attributed to stochastic errors or differences in sample purity. Similarly, we observe ∼96% primary-to-model concordance in copy number calls in the high-cellularity samples. In contrast to previous reports based on gene expression profiles, we do not observe significant differences at the DNA level between *in vitro* compared to *in vivo* models. This study suggests, at a remarkable resolution, the genome-wide conservation of a patient’s tumor genetics in various pre-clinical models, and therefore supports their use for the development and testing of personalized targeted therapies.

## Introduction

The discovery of cancer specific somatic DNA mutations has led to the development of highly effective therapies targeting the corresponding altered protein via monoclonal antibodies or specific inhibitors. These therapies have enhanced activity with a reduced toxicity for the patient in comparison to cytotoxic agents. Recent advances in high throughput sequencing now drives the discovery of actionable mutations in driver genes [1], [2], genes mediating drug sensitivity [3]–[5] or supports new indications for targeted therapies [6]. In 2011, several clinical trials have resulted in the approval of therapies for BRAF^+^ Melanoma (vemurafenib), ALK^+^ Non-Small cell lung cancer (crizotinib) and JAK2^+^ myelodysplasia (ruxolitininb). It is anticipated that the number of clinically approved targeted therapies will increase in the future with our improved ability to discover targets and the effective repurposing of existing drugs for new indications.

Glioblastoma (GBM) is one of the most devastating cancers: it is the most common primary brain malignancy in adults, accounting for over 14,000 deaths per year in the United States [7]. The standard of care includes surgery followed by chemoradiotherapy. Unfortunately, these treatments are rarely curative and the vast majority of tumors recur locally within a few months. A recent integrated multidimensional genomic analysis has shown that the genetic landscape of glioblastoma is rather heterogeneous with 80% of the patients affected in one of three main signaling pathways, *TP53, PIK3CA* and *RB* [8]. Importantly, the specific alterations affect different genes in these pathways through various somatic events such as point mutations, copy number aberrations or transcriptional deregulation. These molecular profiles led to a classification of GBM tumors which have already proven useful in designing more rationalized targeted therapies. An example is the discovery of *IDH1* as a promising new target for younger GBM patients [9], [10]. Current clinical trials in GBM targeting *EGFR, VEGF, PDGFRA* are all leveraging recent molecular genetic information of GBM [11].

The description of the molecular aberrations in tumors has become so comprehensive that we can rationalize the development of novel targeted therapies. Patient derived pre-clinical tumor models are the optimal tools to understand the mode of drug action as well as resistance mechanisms. In most cancers, stable cell lines cultured *in vitro* show a distinct expression profile from primary GBM tumors [12] therefore raising concerns about their validity as clinical models. In GBM studies, neurosphere cultures grown in growth factor supplemented serum-free media are closer to the primary tumor than serum-fed cell cultures [13], specifically from a cellular and transcriptional perspective. Finally, *in vivo* (xenograft) expansion of various glioma cell lines leads to more consistent and more physiologic transcriptional profiles than *in vitro*, with an advantage for intra-cranial over heterotopic mouse xenografts [14]. Therefore, there are significant phenotypic and transcriptional differences between the various tumor models and primary tumors. These differences are seriously undermining our ability to measure and understand candidate drug effects in pre-clinical studies. However, beyond the phenotypic resemblance, the genetic validity of the tumor model is equally important. Indeed most recent therapies in oncology drugs are designed for particular genetic indications, targeting mutated genes, and the corresponding mutations need to be present and maintained in the pre-clinical model to ensure their utility. It is still unclear whether pre-clinical models maintain faithfully the entire DNA mutational profile, including the clonal heterogeneity sometimes found in primary tumors, and can therefore be used to develop and study DNA-guided targeted therapies.

Intra-tumor heterogeneity results from the appearance of distinct mutations in different clones of the tumor, and their subsequent evolutionary selection, as the disease progresses or responds to treatment [15]–[17]. This heterogeneity is a major cause of resistance to standard treatments. Indeed, single agent therapies do not address the molecular heterogeneity, and the process of tumor evolution, which frequently leads to the recurrence of the tumor. Intra-tumor heterogeneity has only been recently studied at the molecular level, through deep sequencing [15], [16], genomic profiling of large tumor sections [18], [19] or even single cell analysis [20]. In GBM, specific investigations of tyrosine kinase receptors amplifications have revealed the presence of independent events in different cells of the same samples [21]–[23]. These observations have important implications on the interpretation of whole-sample genomic studies and their applications to investigate cancer progression and drug sensitivity. For this reason, the development of proper pre-clinical models that can recapitulate and maintain the clonal structure found in primary tumors is critical to generate the knowledge required for the development of meaningful treatment combinations. Because these models, such as cell-lines or mouse xenografts, are generated, grown and maintained in experimental conditions different from the primary tumor physiological conditions, they can themselves undergo clonal selection. An initial selection or a genetic drift can both be detrimental to the utility of these models. The potential variability in mutational profile, including mutation type and prevalence, between primary tumors and pre-clinical models has been only partially investigated. Copy number studies of matched GBM primary and xenograft tumors has provided an estimate of the global genetic validity of the model [24], [25]. However, large genetic differences between a primary GBM tumor and derived model have also been observed. The well studied glioma cell line U87 for example, shows extensive DNA alterations, which likely resulted from *in vitro* clonal selection leading to a mutational profile clearly different from a GBM primary tumor [26]. Similarly, genetic drift has been observed after expansion of clonal cell populations *in vitro* [27]. Elsewhere, it has been observed that *in vitro* growth of GBM cells selects against *EGFR* amplification and mutations, in contrast to *in vivo* xenograft models [28]. In breast cancer, whole genome sequencing of matched primary and xenografts are in good agreement, however there is some evidence of clonal evolution in the xenograft, suggesting that additional work is needed to understand the origin and significance of these differences [29].

Here we describe the results of a whole exome sequencing (WES) of four patient’s primary glioblastoma and their respective tumor models: one neurosphere culture, one laminin cell culture and two xenografts (Figure S1). We identify both somatic mutations and copy number aberrations by comparisons with normal DNA obtained from the patient’s white blood cells. We present an extensive comparison of the primary and model tumor genetic profiles. We develop original analysis methods to perform accurate comparisons and overcome technical variability. Our results illustrate the heterogeneity of the disease from the molecular standpoint and suggest that the pre-clinical models studied maintain their respective parental tumor genetic profiles, regardless of known expression differences [14]. This work therefore confirms, at a resolution superior to previous reports, that pre-clinical models can support laboratory investigations and testing of DNA-guided therapies for the treatment of GBM.

## Materials and Methods

### Human Tumor Collection

Human tissue samples were obtained from 4 newly diagnosed glioblastoma patients under a UCSD Institutional Review Board approved the study. All patients signed a written consent form approved by the Institutional Review Board. No treatment was administered prior to obtaining tissue samples. The samples were de-identified, banked as frozen tissue and used to extract DNA (DNAeasy kit QIAGEN) for the present study. Fresh tumor tissues were used to generate tumor sphere cultures and xenografts as described below.

1. Sample SK01600 was resected from a 57-year-old female presented with a large right frontal mass. The pathology showed classical features of glioblastoma. Molecular biomarkers detected in SK01600 include trisomy of chromosome 7, EGFR amplification (FISH) and overexpression (IHC), PTEN and RB1 hemizygous loss and c-MET gain (+1) (FISH).
2. Sample SK00115 was resected from a 64-year-old male, who presented with a right inferior frontal mass. Pathology showed dense hyper cellularity with astrocytic morphology with small monomorphic and anaplastic cells, florid glomeruloid microvascular proliferation, and pseudopalisading necrosis. Immuno-histochemistry analysis reveals loss of p16, PTEN, and p53 as well as a wild type PDGFR-A. Wild type EGFR and cMyc copy number was confirmed by fluorescent in situ hybridization.
3. Sample SK00102 was resected from a 47-year-old male with a right frontal mass. Pathology showed moderate to high cellularity, widespread microvascular proliferation, geographic zones of necrosis and infiltration into white matter.
4. Sample SK00072 was resected from a 60-year-old male who presented with a left occipital mass. Pathology showed moderate to focally high cellularity, pleomorphic astrocytic tumor cells, necrosis, and infiltration into white matter. Molecular biomarkers detected in SK00072 include trisomy of chromosome 7, loss of chromosome 10, EGFR amplification (FISH) and over-expression (IHC), RB1 and CDKN2A deletions (FISH), PDGFR-A and –B overexpression (IHC).

### Short-term Tumor Cultures

SK01600 and SK00115 GBM cells cultures were derived from above described primary GBM tissues as follows. Tumor specimens were washed in HBSS and mechanically minced, then dissociated using the MACS Neural Tissue Dissociation Kit (Miltenyi). Cells were subsequently washed, filtered through a 40-µm strainer and plated in low-attachment plates and grown as neurosphere (SK01600) in NeuroCult NS-A proliferation media (Stemcell Technologies) supplemented with 10 ng/mL rhbFGF (StemGent) and 20 ng/mL rhEGF (Stemcell Technologies) [13]. Alternatively, tumor cells were plated in laminin-coated plates (SK00115) and grown in adhesion using the above-indicated media [30]. Tumor cells were incubated at normal oxygen levels, at a temperature 37.0°C and 5% CO2. Samples were collected at passage 6 (SK01600) and passage 3 (SK00115). The DNA was extracted using DNAeasy DNA extraction kit (QIAGEN).

### Xenograft Model

SK00072 and SK00102 primary GBM tissues were directly passaged in vivo as mouse xenografts. Fresh tumor tissues were washed in HBSS and mechanically minced. Tissue aggregates were suspended in HBSS and mixed one to one with Matrigel (BD Biosciences) for injection. Six to eight week-old immuno-compromised NSG mice (The Jackson Laboratory) were injected at the flank [31], [28]. Tumors were removed when size reached 1–1.5 cm^3^. DNA extraction was performed using DNAeasy DNA extraction kit (QIAGEN). For in vivo tumor maintenance, part of the tumor was mechanically dissociated as described above and reinjected subcutaneously into mice. The xenografts studied were collected at the first passage in vivo. All in vivo experiments were conducted under a protocol approved by UCSD IACUC (Institutional Animal Care and Use Committee).

### Data Generation

Three microgram of genomic DNA were fragmented to ∼150 bp (Covaris S2, Covaris, Inc., Woburn, MA). The fragmented DNA was then subjected to SOLiD library preparation and SureSelect Exome capture by hybrid selection following the manufacturer’s instruction (see Methods S1 for details). The captured exome libraries were sequenced for a single end on the SOLID 3.0 instrument (Applied Biosystems) for 50 cycles for the forward read and 35 cycles form the reverse read. We generated more than 250M reads per sample (Table S9). The sequencing data is available at the NCBI short read archive database accession number SRA049073.1.

### Data Analysis

The reads were aligned to the human hg19 reference genome using Bioscope 1.3.1 (Life Technologies, Carlsbad, CA) followed by post-alignment improvements steps implemented in Picard or GATK [32]. The alignment quality is summarized in Figure S2 and Table S1. For patients with xenografted samples, the reads were also aligned to mm9 mouse reference genome and only reads aligning the human genome specifically or with a better alignment based on pairing and matching information were kept for subsequent analysis. The germline, somatic and loss of heterozygosity variants were called using VarScan 2.5.5 [33], using default filters and were annotated using the SeattleSeq server. Variants with a significantly lower alternate allele base quality were likely false positive were identified using mixture modeling (MCLUST) [34] and discarded. Low confidence somatic variants were removed by applying a minimum right-tailed Fisher exact P-value of 0.05 as determined by VarScan. We removed germline variants incorrectly called somatic, by requesting a maximum of 5% alternate allele frequency in the normal DNA. Germline Insertion/deletions (indels) were called with more stringent criteria, requiring 10× coverage, 3 reads supporting the variant and less than 5% mutant reads in the germline. After calling all somatic mutations, the mutant allele frequency in the primary and model tumors were compared and assessed via Fisher Exact test, with a false discovery rate of 0.05, as estimated from a permutation test. The difference in the mutant allele frequencies between the primary and model tumors was also used to estimate the amount of normal DNA contamination in the primary, assuming a pure model sample (Methods S1). Copy number aberrations were called from the exome sequencing data using ExomeCNV method [35], after correction for GC content (Figure S3 and Methods S1). Large chromosome arm copy number aberrations were called when >20% of the targeted base pairs of a chromosome arm were consistently called as CNA. Focal amplifications were called from high confidence (HC) calls. HC amplified (respectively deletions) segments are segments with a logR ratio higher (respectively lower) than the 95^th^ (respectively 5^th^) percentile of the logR ratio of copy neutral segment. More details are available as supplementary method (Methods S1).

### Genotyping Array Data Analysis

Omni2.5-Quad IDAT intensities were processed to genotypes using GenomeStudio (version 2010.3) using default cluster positions (HumanOmni2.5-4v1_D.egt) and the default GenCall score cutoff of 0.15 for Infinium arrays. Genotypes were exported in reference genome PLUS orientation (build hg19) based on HumanOmni2.5-4v1_D.bpm. We converted 1000 Genomes Project SNPs (kgp identifiers) to rsIDs by matching chromosome, position, and alleles in dbSNP132. We restricted SNPs to those that are present and biallelic in dbSNP132, and did not evaluate indels. We excluded 17,959 SNPs that were present in duplicate on the chip, 11,536 SNPs that had more than 2 alleles in dbSNP, and 405,516 SNPs, which were not reported in dbSNP132. This resulted in a total of 2,016,730 SNPs. Due to questionable strand orientation, a previously reported problem, we additionally filtered 61,690 A/T and C/G SNPs that overlapped with the targeted region of the sequencing.

## Results

We performed whole exome capture using hybrid selection [36] of 12 samples (4 blood, 4 primary and 4 models) from 4 GBM patients (SK01600, SK00115, SK00072 and SK00102). High-throughput sequencing resulted in ∼69% of the targeted bases covered at 10× or more, for an average on-target coverage of 59× across all 12 samples, therefore allowing accurate base calling at the majority of the coding portion of the genome (Table S2). We first assessed the quality of the resulting calls by analyzing germline single nucleotide variants (SNV) comparing the results of sequencing and microarray genotyping at 62,550 positions investigated by the two methods. Of those, 52,905 were confidently called by sequencing of patient SK00072 germline DNA, passing our quality review. Ninety-seven percent of them had a consistent call between the two methods. Across all four patients, we estimated that ∼90% of the germline SNVs identified are present in dbSNP(132) (Table S3), which is slightly lower than expected (95%) for Caucasian patients [32]. A close inspection of the novel SNVs reveals a bi-modal distribution of the quality score of the alternate allele, contrasting with the single distribution at known SNPs (Figure S4). This indicates that a subset of the novel SNPs is of lower confidence and possibly resulting from sequencing errors. We separated the two distributions using normal mixture modeling [34]. The resulting set of high quality SNPs are now ∼95% in dbSNP, and their transition to transversion ratio is ∼3.1 (Table S3), closer to expected [32], therefore indicating an improvement in the accuracy of the SNV calls. Learning from this analysis of germline variants, we subsequently applied this strategy to filter all somatic calls.

### Primary Tumors Mutational Landscape

**Figure 1:**
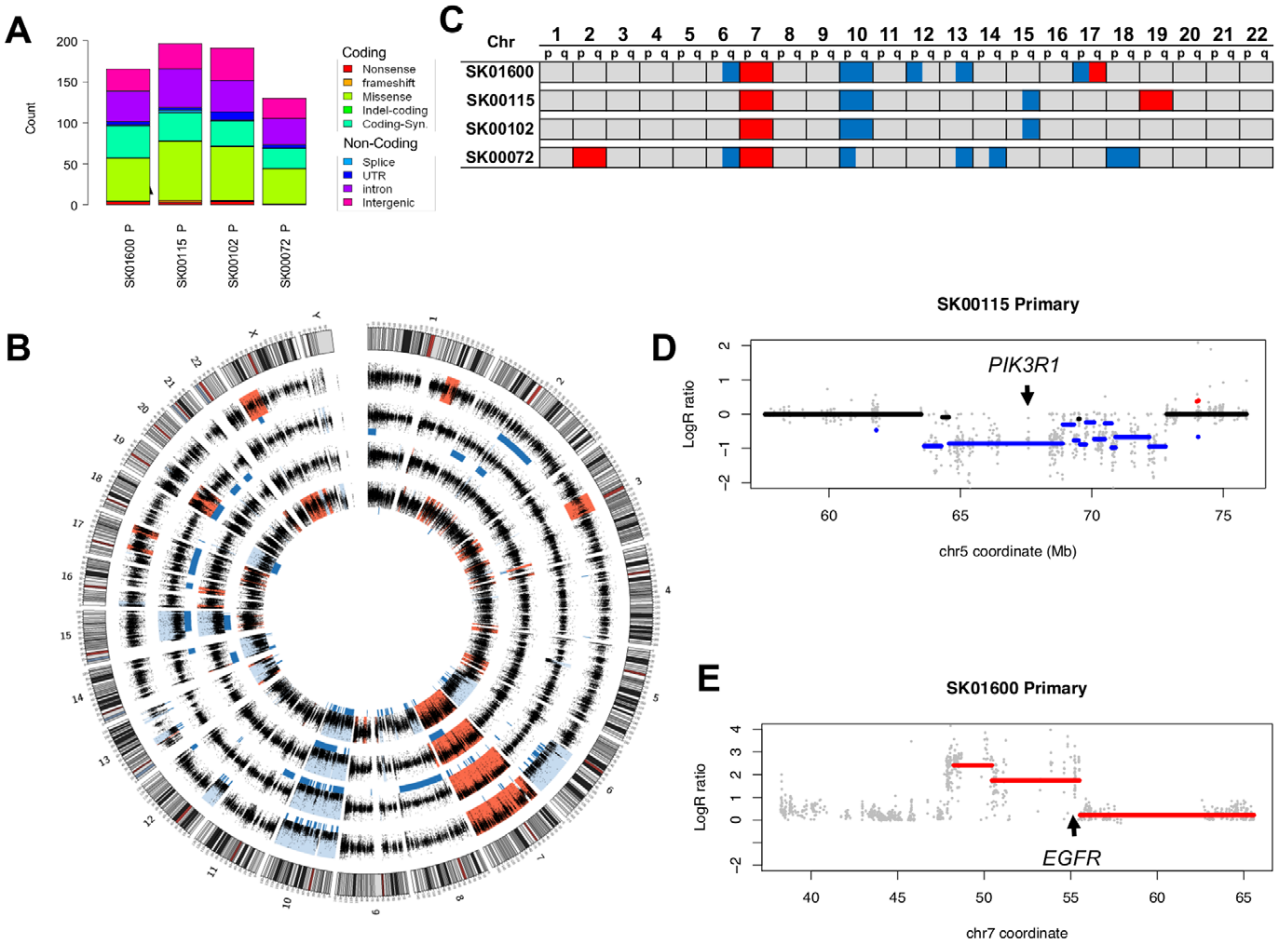
Mutational Landscape of the primary tumors. (**A**) The cumulative distribution of the somatic mutations identified on the targeted exons of the four patients primary tumors is reported as a function of their class and predicted protein changes. (**B**) Circular diagram [48] representing all 23 chromosomes and their cytogenetic map (outer circle, grey scale bands and red centromeres). The logR tumor/normal coverage ratios (black dots) and the inferred CNA (red: amplification, blue: deletion, blue bars: Loss of Heterozygosity) identified in the 4 primary tumors (from outer to inner circle: SK01600, SK00115, SK00102, SK00072) using whole exome sequencing data are represented. (**C**) Chromosome-arm level copy number aberrations are observed in the 22 autosomes when >20% of a chromosome arm is reported as deleted (blue) or amplified (red). (**D**) A focal deletion of ∼10 Mb (set of blue segments) including a large (4.3 Mb) CNA segment affects *PIK3R1* gene in SK00115 primary tumor. The LogR ratio of tumor/normal coverage (x axis) at each exon capture probe (grey dots) allows the identification of DNA segments deleted (blue bars) or amplified (red bars). (**E**) Similar to (D), a focal amplification of *EFGR* containing segment (red) is identified in addition to the chromosome 7 trisomy in patient SK01600 primary tumor. Some segments may appear to overlap as a result of the plotting resolution.

We compared the variant calls between tumor and normal DNA [33] restricting our analysis to the positions located on the capture targets. We identified a total of 682 somatic mutations across all patients, ranging from 130 to 191 per patient (Table S4). Of these, 384 are located in coding exons or a predicted splice site with 234 (61%) missense, 12 (3%) nonsense, 3 (<1%) frameshift, 1 (<1%) in-frame deletion, 5 (1%) splicing and 129 (34%) synonymous mutations (Figure 1A). This distribution leads to a non-synonymous to synonymous ratio of 1.98 consistent with the positive selection of driver mutations. These numbers and distributions are also in agreement with the mutational profile observed in exomes of GBM and other solid tumors [8], [37]. In order to determine which of these mutations are more likely to play a role in GBM progression, we used information from larger repositories such as COSMIC [38] or TCGA [8]. Ten of the 250 non-synonymous mutations have been previously identified in cancer samples (Table 1), among these, three *PTEN* mutations in two patients were previously found in gliomas and three *TP53* mutations in one patient were previously identified in various cancer types [38]. We also identified one patient with an EGFR-C326S mutation, a position previously seen mutated in glioblastoma [39], as well as one patient with a NRAS-Q61K mutation, common in melanoma but never seen in gliomas. Expanding our investigation to 2,850 genes known to be mutated in gliomas [8], we note a total of 59 non-synonymous or splice-site mutations in 53 genes (Tables S5 and S6). Apart, from *PTEN*, *GPR98* is the only recurrently mutated gene. This gene spans more than 600 kb and mutations in its sequence are more likely to be passenger. Therefore, except for mutations in *PTEN*, the four patients seem to have mostly divergent sets of mutations contributing to the genetic make-up of their cancer.

**Table 1:** Somatic non-synonymous mutations observed in the primary tumors and overlapping with known COSMIC (v55) entries.

Patient	NCBI37	Alleles	Number of reads (ref:mut)		Gene	Type	Codon change	Cancer Type
		Reference/Mutant	Germline	Primary^1^				
SK01600	chr7∶55223610	G/C	7∶0	32∶18	EGFR	missense	C326S	Glioma 2
SK00115	chr10∶89692792	C/A	217∶0	43∶45	PTEN	missense	D92E	Glioma
SK00115	chr17∶7577143	AGT/−	14∶0	7∶4	TP53	In frame deletion	L265	Large intestine
SK00115	chr17∶7578205	C/A	16∶0	17∶7	TP53	missense	S215I	Various non gliomas
SK00115	chr17∶7578458	G/A	18∶0	9∶3	TP53	missense	R158C	Glioma^2^
SK00102	chr1∶115256530	G/T	96∶0	82∶25	NRAS	missense	Q61K	Various non gliomas
SK00102	chr10∶89685288	T/A	10∶0	2∶2	PTEN	missense	H61Q	Glioma^2^
SK00102	chr10∶89711875	G/A	123∶0	34∶42	PTEN	missense	G165R	Glioma
SK00102	chr16∶3790512	G/A	13∶0	19∶6	CREBBP	nonsense	R1303*	Various non gliomas
SK00072	chr1∶159504907	C/G	27∶0	36∶14	OR10J5	missense	K297N	Various non gliomas

1 Can includes normal DNA contamination and effect of copy number.

2 The same position but not the same mutation was found in Glioma.

Large chromosomal aberrations such as chromosome 7 trisomy or a loss of chromosome 10 are an important characteristic of glioblastoma mutational landscape. Although traditionally assayed through cytogenetic assays or Comparative Genomic Hybridization (CGH) and more recently with next generation sequencing [35], [40], copy number aberrations (CNAs) can also be identified via exome sequencing strategies, using notably coverage differences, between tumor and normal DNA as well as evidence of loss of heterozygosity. Applying ExomeCNV [35], a segmentation strategy to evaluate the copy number and Loss of Heterozygosity (LOH) status of consecutive exons, we were able to call 32 large (chromosome arm level) CNAs in the four primary tumors (Figure 1B, 1C). All 4 patients showed a loss of chromosome 10 and an amplification of chromosome 7. Half of the patients also show evidence of a loss of one allele in chromosome 6q, 13q or 15q. Deletions of 12p, 14q, 17p or amplification of 2p and chromosome 19 were also observed each in a single case. The majority of these large CNAs are consistent with the most frequently recurring CNAs in glioblastoma [8]. We also identified 23 high confidence focal CNAs (8 amplification and 15 deletions) in regions outside of large CNAs. Six of them encompass genes of the Cancer Gene Census [41] (Table S7). Patient SK00115 shows a 4.3 Mb deletion around *PIK3R1* (Figure 1D). *PIK3R1* has been identified as a candidate cancer driver gene and is mutated in ∼9% of GBM patients [39], [8], but the loss of one allele, as seen here, has not been reported. Other CNAs deleted or amplified more than 2 fold in one or more sample are affecting 72 cancer genes and correlate well with array CGH diagnostic results obtained on three patients (Table S8). Notably, we could verify the 4-6-fold amplification of *EGFR* locus in SK01600 primary tumor, encompassing a 5 MB segment (Figure 1E). This focal high-level amplification occurs in 40% of glioblastoma conjointly with the more common trisomy of chromosome 7. It is important to note that, in contrast with CGH and whole genome sequencing strategies, whole exome sequencing can introduce some bias in the estimation of CNAs: exons are not evenly distributed along the genome, which can lead to issues in resolving focal amplifications using whole exome sequencing coverage data [40]. Our results suggest however that exome-based CNA calls are a good indicator of the presence of CNAs genome-wide and can therefore be used in cases where the amount of available DNA is scarce, a frequent situation in oncology translational studies.

Taken together our results reveal common molecular markers of GBM primary tumors, including nucleotide substitutions, small insertions and deletions as well as CNAs. Our results of both gene mutations and copy number alterations illustrate the heterogeneity observed across 4 patients, which is typical of the diversity of glioblastoma seen in the clinic. Some of these mutations are considered clinically actionable, such as alterations in the PI3K pathway (loss of *PTEN* or *PIK3R1*, amplification of *PIK3CA*) for which targeted therapies are currently in clinical trials in several cancer types. Having established a comprehensive mutational profile of the primary tumor, we can now use the same, high-resolution assessment, to study the maintenance of this profile in the corresponding pre-clinical models.

### Comparison to the Tumor Model Mutational Profile

**Figure 2:**
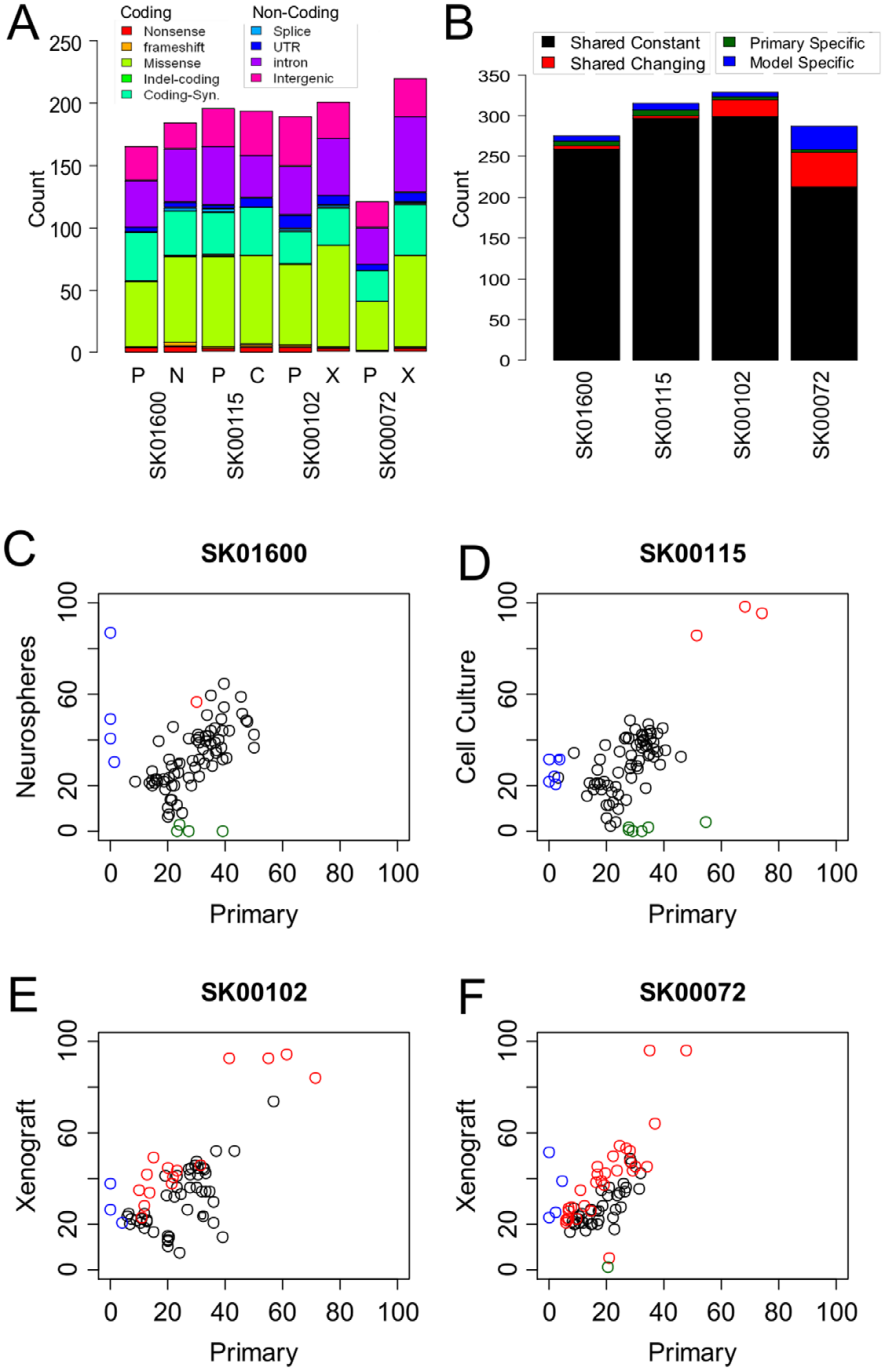
Comparative evaluation of the somatic mutations between primary and model tumors. (**A**) The cumulative distribution of the somatic mutations identified on the targeted exons of the four patients’ primary tumors (P) as well as tumor models (N: Neurospheres, C: Cell culture, X: Xenograft) is reported as a function of their class and predicted protein changes. The mutations were identified after excluding mouse reads from patients’ SK00102 and SK00072 data. (**B**) A statistical comparison of the somatic mutations called between primary and model identifies shared mutations at constant mutant allele frequencies (black), shared mutations with changing mutant allele frequency (red) as well mutations specific to the primary (green) or the tumor model (blue). (**C–F**) Mutant Allele frequency differences between the primary tumor (x axis) and the model tumor (y axis) of patient SK01600 (**C**), SK00115 (**D**), SK00102 (**E**), SK00072 (**F**) at all positions identified as somatically mutated in either sample and covered by ≥30 reads. Mutations are classified as shared with constant frequency (black), with changing frequencies (red), specific to the primary tumor (green) or to the tumor model (blue).

We applied the strategy described above for the primary tumors to identify somatic mutations in each tumor model derived from the four patients. The number of mutations in the SK01600 cells and SK00115 *in vitro* cultures was 184 and 194 respectively in close agreement with the findings in the primary tumor (165 and 196 respectively –Figure S5). In contrast, we noticed 1.8 and 3.6 fold excess in the number of mutations in the two xenografted tumors, SK00102 and SK00072 respectively. We suspected that mouse DNA contaminated the xenograft tumor samples, which led to their unspecific capture and alignment to the human genome, especially at genes of strong orthology. Using mouse to human alignment comparison, we were able to identify the most likely species of origin of each sequenced fragment (Methods and Figure S6). As expected, the resulting filter does not significantly change the number of somatic mutations identified in SK00102 and SK00072 primary tumors – from 191 and 130 to 189 and 121, respectively – whereas it significantly decreases the number of somatic mutations in the xenograft samples – from 338 and 468 to 201 and 220 respectively (Table S9). Thus, in all the subsequent analysis, we used reads filtered for murine contamination for all SK00102 and SK00072 samples (germline, primary and xenografts). Comparing the total number of somatic variants identified in all 8 samples, we note that SK00072 primary tumor shows fewer somatic mutations (Figure 2A) and a reduced mutant allele frequency (p<7 10^−4^) (Figure S7). These observations suggest that SK00072 primary tumor DNA sample contains normal DNA leading to a reduced sensitivity to detect somatic mutations. This conclusion is confirmed by the histological analysis of the tissue, indicating parenchymal infiltration within this tumor specimen (Methods). Contamination of the tumor DNA with normal DNA is a recurrent challenge for the sensitive detection of somatic mutations via high throughput sequencing. Therefore, it is important to know whether the derivation of pre-clinical models, in addition to preserving the tumor clonal heterogeneity, can have a purifying effect by selecting tumor cells only.

The total number of somatic mutations is in agreement between all three types of tumor models and their respective tumor of origin, suggesting an equivalent mutational load and the absence of hyper-mutator phenotype acquired during the derivation of the model. In order to refine this vision, and detect rare somatic differences between primary and model, we implemented a strict statistical comparison of the fraction of mutant allele supporting reads at all positions identified as somatic mutations in each set of matched samples. We were able to distinguish between shared mutations showing no significant changes in frequencies (referred to as shared constant), shared mutations with a significant change in frequency (referred to as shared changing), and mutations identified only in the model or the primary (referred to as unique), at a false discovery rate of 0.05. On average across all 4 pairs, 98% of the mutations identified in the primary were shared with the model (Figure 2B). Reciprocally, only ∼2% of the mutations identified in the model were unique and not found in the primary, with the exception of patient SK00072 for which 11% (23/213) of the mutations are unique to the model. This result is not surprising given the lack of sensitivity to detect somatic mutations in SK00072’s primary tumor due to normal DNA contamination. This observation supports the idea of a purifying process during the derivation of the xenograft, either through the preparation of the sample or during its expansion *in vivo*. For the remaining three pairs of samples, a discrepancy of ∼2% between primary and model is within the range observed when comparing mutations detected in control split-sample experiments (Table S9), and below our FDR threshold, therefore pointing to a systematic bias rather than true genetic differences.

Out of the 1005 mutations shared between primary and model, 293 were covered by 30 reads or more in both samples and showed a high correlation in mutant allele frequency between primary and model (r>0.7) (Figure 2C–F). Furthermore, 48 out the 49 of the mutations covered at 30× with a significant change in mutant allele frequency (FDR = 0.05) show a unidirectional change, a modest enrichment in the model, suggesting a higher purity of these samples when compared the primary rather than true allele frequency differences due to clonal selection. These results suggest therefore the absence of strong clonal selection using either *in vitro* or *in vivo* models. Using the differences in allele frequency, and assuming the purity of cancer cells in the tumor models, we can establish that the primary tumors were contaminated with 9%, 11%, 25% and 41% of normal DNA in patient SK01600, SK00115, SK00102 and SK00072, respectively, which is consistent with the reduced sensitivity in detecting mutations in SK00072 primary tumor.

**Figure 3:**
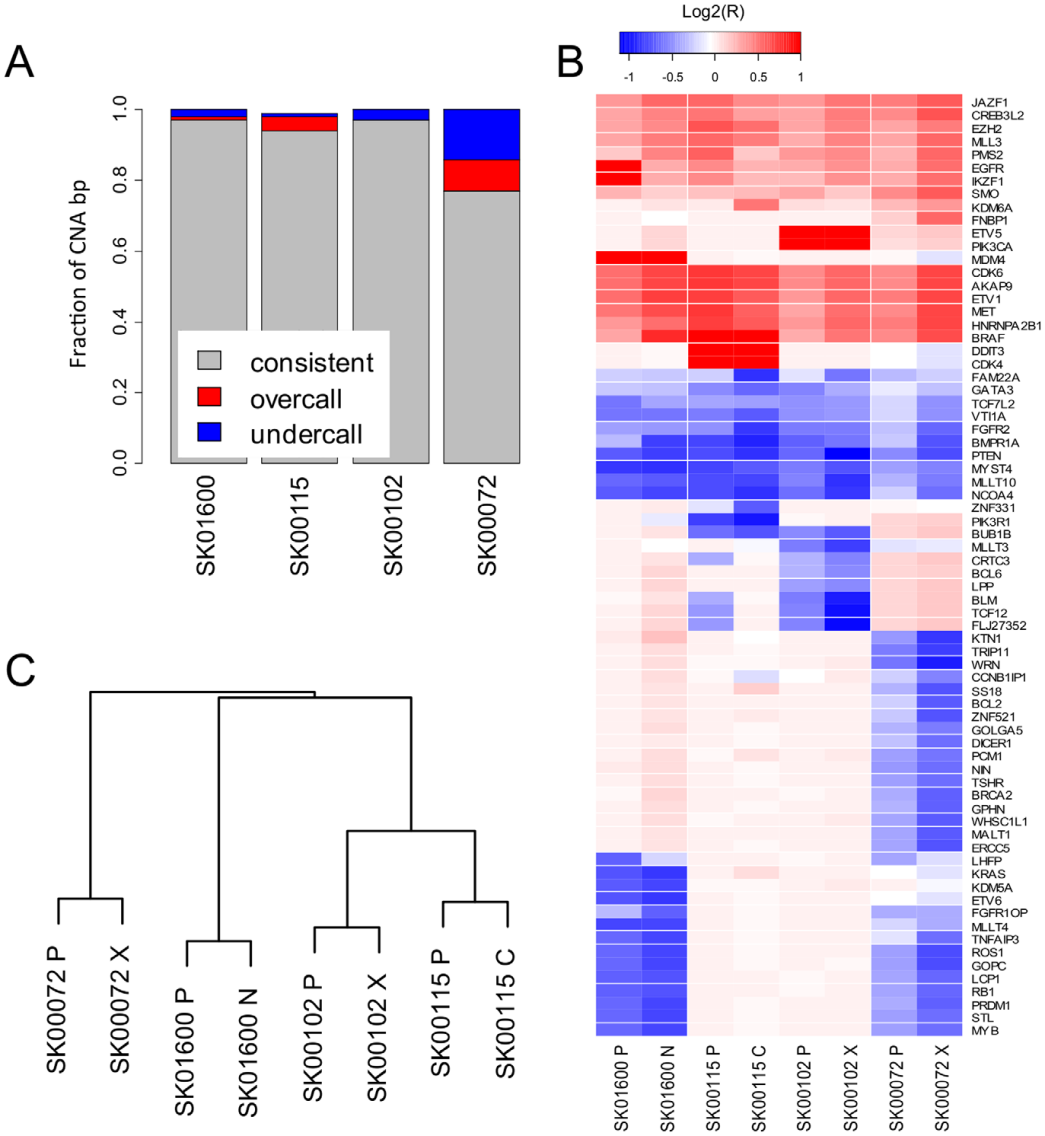
Comparative evaluation of the CNAs between primary and model tumors. (**A**) The evaluation of the copy number status at all base pairs called in high-confidence CNA segments in both primary and model tumors identifies positions with a consistent (grey), lower (blue) or higher (red) copy number call in the model when compared to the primary tumor (Table S10). (**B**) Average copy number status (blue-red color scale, log2 ratio) at 72 genes of the cancer gene census showing more than 2 fold copy number difference in one or more sample. (**C**) Euclidian distance based dendrogram classifying the 8 tumor samples using the logR ratio of high-confidence CNA called in one or more sample.

We next evaluated whether CNAs were conserved between primary and model. Restricting the primary-model comparison to high confidence CNA calls, we observed that 97, 94 and 97% of the base pairs in CNAs are consistently called between primary and model in samples SK01600, SK00115, SK00102, respectively (Figure 3A and Table S10). In contrast only 77% of the high confidence CNAs base pairs show this level of consistency between the two SK00072 samples, while 14% are called in the primary at a lower copy number than in the model. This result is consistent with the presence of normal cells in SK00072 primary tumor, which affects the sensitivity of CNA detection. Although the global landscape of structural variants is important to study the mechanisms of cancerogenesis and clonal selection, our ability to interpret the biological consequences of CNAs is limited to the coding portion of the genome, where gain and losses of specific alleles have a frequently demonstrated oncogenic potential. In order to validate our method for the accurate estimation of more biologically significant CNAs, we performed a specific inspection of the copy number status at 450 genes from the cancer gene census [41]. We could identify 72 genes with a copy number change of more than 2 fold in one or more samples (Figure 3B). Consistent with the previous results, there is a very strong correlation between the copy number observed in the primary and in the tumor model. Interestingly, the copy number estimation in SK00072 primary tumor is lower than in its matched xenograft, again highlighting how contamination of the primary sample with normal DNA can underscore the sensitivity of the mutational analysis. We observed the strong amplification of *EGFR* and neighboring gene *IKZF1* in the primary tumor of patient SK01600, but it seems to be partially lost in the matched neurosphere culture. This result is consistent with the frequent loss of *EGFR* amplification in serum-fed culture [28], as well as growth factor supplemented serum-free culture [42].

Overall, using genome-wide high-confidence CNA calls as a molecular signature, we observed that primary and tumor models are more related to one another that to another patient sample (Figure 3C). We do not observe a closer relationship between *in vivo* models and primary than between *in vitro* model and primary therefore showing that, from a genetic perspective, *in vitro* and *in vivo* models are both faithfully matching the primary tumor.

## Discussion

High-throughput DNA sequencing now offers the opportunity to obtain a detailed molecular profile of a biological sample such as the ones we studied here. This recent technology has been evolving at fast pace, and some systematic errors and samples preparation difficulties can make their use challenging. This is especially true for studies aimed at comparing longitudinal samples, between primary tumor and relapse, or between primary and tumor-model. Systematic errors, often platform dependent, can lead to false positive rates sometimes as high as 10% [43]. We present here novel analytical methods that can increase our confidence in the mutations detected through exome sequencing, by identifying mouse reads, correcting for mutant allele base quality, accounting for GC bias in CNA calls or performing a strict statistical comparison between two samples instead of relying on simple identification of the mutants. Through these improvements, our results support the global maintenance of the primary tumor genetic profile in the chosen pre-clinical model.

We demonstrate that intra-tumor clonal heterogeneity is conserved in the various models. The maintenance of tumor heterogeneity is important to understand the mechanism of resistance occurring in the patient during therapy. Recent study of post-relapse leukemia have shown the presence of the resistant clone in earlier samples [15]. Such studies have yet to be performed in glioblastoma, and xenografts would be an advantageous model for this, as the tumor can be isolated at different stages of the progression. Similarly, the number of passages of *in vitro* and *in vivo* models is thought to cause important genome remodeling, principally through large structural rearrangements and polyploidy. Model cell lines, maintained in the laboratory for decades, such as U87, show highly remodeled genome, with little in common with the genome of primary tumors. Stable late passage models tend to mimic more closely the biology of the primary tissue, but early passage models are highly valuable for identifying the optimal targeted therapy within the lifespan of the patient [44]. Therefore, although we did not strictly address the genetic drift of the tumor through passages, our results suggest that a moderate number of passages (1 for *in *vivo, 3 to 6 for *in *vitro) do not have detectable consequences on their genomes. One exception seems to be the maintenance of *EGFR* copy number *in vitro,* which is affected by the presence of EGF in the medium. As more inhibitors of growth factors such as nilotinib for *PDFGRA*, are being tested in clinical trial, it is crucial to carefully select the laboratory conditions in which pre-clinical experiments are being performed and to ensure that the presence of the marker can be maintained *in vitro*.

Molecular profiling of patients and patient-derived samples has become a central part of personalized medicine. Several centers are promoting the clinical sequencing of patients’ samples to guide treatment [45]. As an increasing number of targeted therapies are approved, drug resistance will become increasingly problematic and repeated molecular profiling on relapse biopsies will be needed to choose the appropriate second line of therapy and hopefully convert cancer in a manageable disease under surveillance [46]. For these reasons, that directly impact the care of the cancer patients, the availability of representative pre-clinical model to study drug sensitivity and resistance becomes crucial. Two recent drug screening studies of fully characterized cell lines illustrate the utility of combining genomic and pharmacological information [4], [3]. This approach has limitations, as it does not recreate the micro-environmental niche in which the primary tumor resides and grows and that may as well affect treatment response [47], [30]. Nonetheless, our results indicate that both patient-derived *in vitro* cultures and *in vivo* xenografts represent robust pre-clinical models systems reflecting the genomic diversity of primary GBMs, and highlight their utility in defining tumor genetics predictors of drug response and drift of tumors from selective pressures (e.g. treatments). Hence, ensuring our comprehensive understanding of the molecular forces at play in these models, will favor the successful translation of these discoveries to the clinical care where molecular profiling will become standard.

## Supporting Information

Figure S1 **Experimental Design.** The matched blood and primary tumor’s DNA from 4 patients were analyzed in addition to the patient derived neurospheres (SK01600), laminin cell culture (SK00115) or mouse xenografts (SK00102 and SK00072).
(JPG)

Figure S2 **Sequencing Quality Assessment.** **(A)** The Reads were sequenced on SOLiD4 instrument and aligned to the reference genome using BioScope. The duplicate reads were identified using Picard MarkDup and custom scripts (Methods). **(B)** Coverage depth cumulative distribution for all 12 samples (matched germline, primary tumor, and tumor model). **(C)** Capture enrichment specificity. The fraction of bases sequenced on or near (+/−250 bp) the Agilent SureSelect 50MB kit targets is indicated (Table S12).
(JPG)

Figure S3 **SK00115 tumor model shows GC induced bias in the coverage distribution.** Normalized average coverage per GC% of targets for all four patients. Germline (black), primary tumor (red), and tumor models (blue) are displayed.
(JPG)

Figure S4 **Alternate allele’s base quality score filtering. (A)** Distribution of the average alternate allele’s base quality score for germline variants present in dbSNP132. **(B)** Same as (A) for novel germline variants. **(C)** Variants are filtered out (red) when they belong to the lower quality distribution as determined by mixed model deconvolution.
(JPG)

Figure S5 **Identification of somatic mutations in the tumor models. (A)** The number and distribution of mutations in the tumor models matches the primary except for xenograft samples, suggesting mouse DNA contamination. **(B)** The total number of somatic mutation before filtering of the mouse reads (grey) and after filtering of the mouse reads (black).
(JPG)

Figure S6 **Filtering of the Mouse contaminating reads.** A succession of filters (green arrow: pass, red arrow: do not pass) compares pairing information as well as matching score to determine the species of origin of each read. (*) *Match score (M) = # of Matches - # of Mismatches.*
(JPG)

Figure S7 **SK00072 primary tumor shows a significantly lower mutant allele frequency.** **(A)** Distribution of the mutant allele frequency at mutations shared between primary and model. **(B)** Student T-test p-value (red scale –log10 (P-value)) of the 6 possible comparisons from (A), showing SK00072 as significantly lower mutant allele frequency.
(JPG)

Table S1 **Sequencing read mapping statistics.**
(XLSX)

Table S2 **Target Coverage Statistics.**
(XLSX)

Table S3 **Quality of the germline coding variants identified on target.**
(XLSX)

Table S4 **Distribution of the somatic variants by sample and class.**
(XLSX)

Table S5 **Non-synonymous or splicing coding mutations in genes mutated in gliomas samples in the COSMIC database.**
(XLSX)

Table S6 **Number of non-synonymous and splice-site somatic mutations identified in each patient in genes known to be mutated in glioma in the COSMIC database.**
(XLSX)

Table S7 **High confidence focal copy number events detected outside of large CNA regions.**
(XLSX)

Table S8 **Array CGH results obtained from patients SK00072, SK01600 primary tumors and SK00102 xenograft derived neurosphere cultures.** (ND: not determined).
(XLSX)

Table S9 **Comparison of somatic mutations called in the primary tumor or in the tumor model.**
(XLSX)

Table S10 **Contingency table comparing the amount of DNA sequence (bp) in high confidence copy numbers segments called as copy number neutral (Neu), deleted (Del) or amplified (Amp) status or where copy number could not estimated with confidence (NC).**
(XLSX)

Table S11 **Total number of variants detected by VarScan on the targeted regions.**
(XLSX)

Table S12 **Exome Capture Specificity.**
(XLSX)

Methods S1 **Additional description of material and methods used or developed in this study.**
(DOCX)
