# Supplementary figures and images for: High-Resolution Mutational Profiling Suggests the Genetic Validity of Glioblastoma Patient-Derived Pre-Clinical Models
Source: PLoS One. 2013 Feb 18;8(2):e56185. doi: 10.1371/journal.pone.0056185 (PMC3575368; doi:10.1371/journal.pone.0056185)

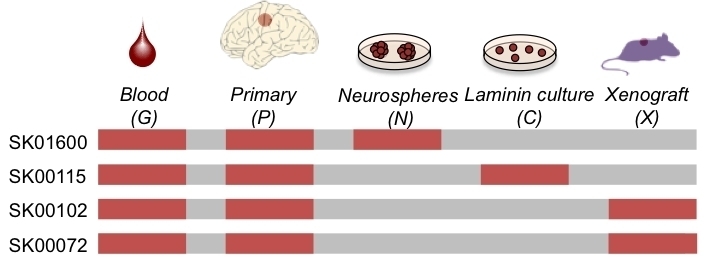

Supplement: Figure S1 — Experimental Design. The matched blood and primary tumor’s DNA from 4 patients were analyzed in addition to the patient derived neurospheres (SK01600), laminin cell culture (SK00115) or mouse xenografts (SK00102 and SK00072). (JPG) [file pone.0056185.s001.jpg]

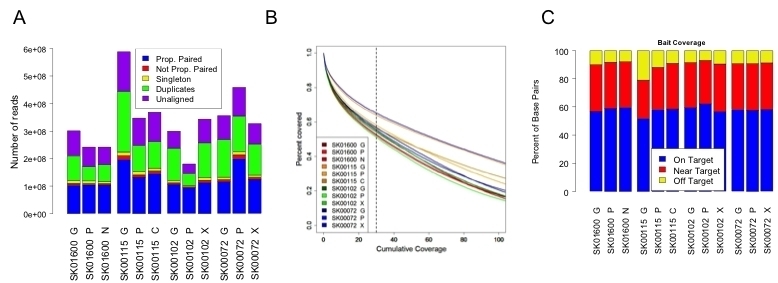

Supplement: Figure S2 — Sequencing Quality Assessment. (A) The Reads were sequenced on SOLiD4 instrument and aligned to the reference genome using BioScope. The duplicate reads were identified using Picard MarkDup and custom scripts (Methods). (B) Coverage depth cumulative distribution for all 12 samples (matched germline, primary tumor, and tumor model). (C) Capture enrichment specificity. The fraction of bases sequenced on or near (+/−250 bp) the Agilent SureSelect 50MB kit targets is indicated (Table S12). (JPG) [file pone.0056185.s002.jpg]

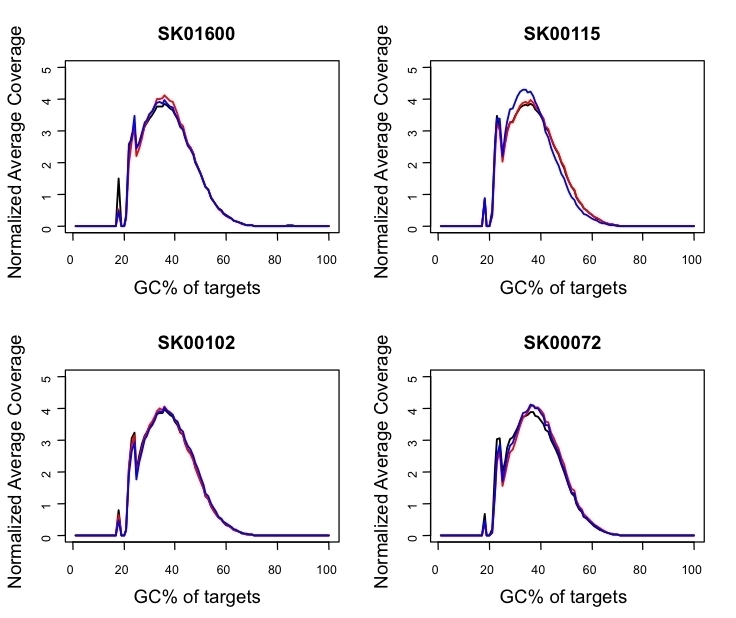

Supplement: Figure S3 — SK00115 tumor model shows GC induced bias in the coverage distribution. Normalized average coverage per GC% of targets for all four patients. Germline (black), primary tumor (red), and tumor models (blue) are displayed. (JPG) [file pone.0056185.s003.jpg]

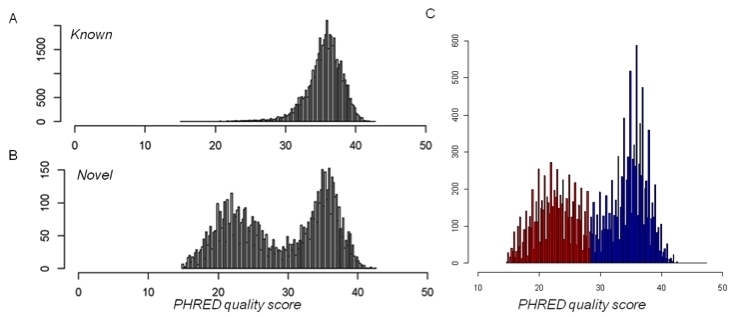

Supplement: Figure S4 — Alternate allele’s base quality score filtering. (A) Distribution of the average alternate allele’s base quality score for germline variants present in dbSNP132. (B) Same as (A) for novel germline variants. (C) Variants are filtered out (red) when they belong to the lower quality distribution as determined by mixed model deconvolution. (JPG) [file pone.0056185.s004.jpg]

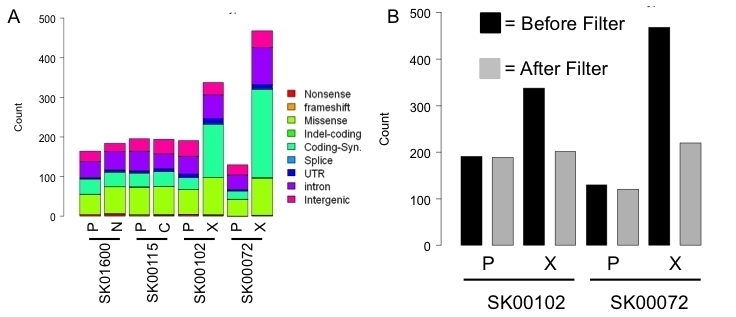

Supplement: Figure S5 — Identification of somatic mutations in the tumor models. (A) The number and distribution of mutations in the tumor models matches the primary except for xenograft samples, suggesting mouse DNA contamination. (B) The total number of somatic mutation before filtering of the mouse reads (grey) and after filtering of the mouse reads (black). (JPG) [file pone.0056185.s005.jpg]

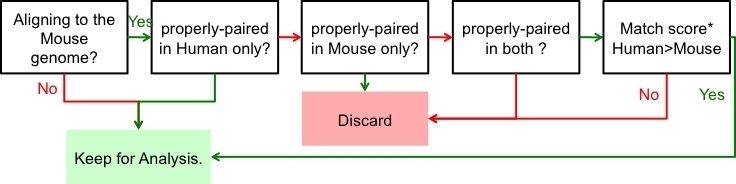

Supplement: Figure S6 — Filtering of the Mouse contaminating reads. A succession of filters (green arrow: pass, red arrow: do not pass) compares pairing information as well as matching score to determine the species of origin of each read. (*) Match score (M) = # of Matches - # of Mismatches. (JPG) [file pone.0056185.s006.jpg]

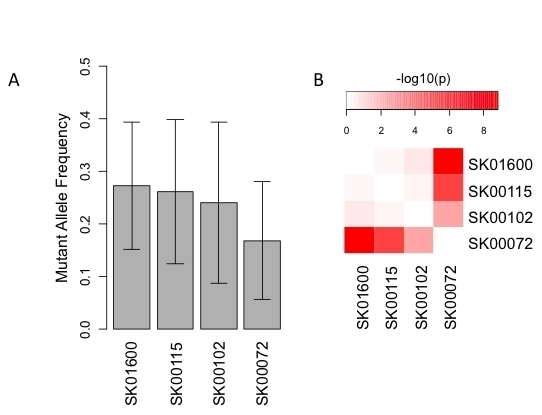

Supplement: Figure S7 — SK00072 primary tumor shows a significantly lower mutant allele frequency. (A) Distribution of the mutant allele frequency at mutations shared between primary and model. (B) Student T-test p-value (red scale –log10 (P-value)) of the 6 possible comparisons from (A), showing SK00072 as significantly lower mutant allele frequency. (JPG) [file pone.0056185.s007.jpg]
