# Supplementary material for: High-Resolution Mutational Profiling Suggests the Genetic Validity of Glioblastoma Patient-Derived Pre-Clinical Models
Source: PLoS One. 2013 Feb 18;8(2):e56185. doi: 10.1371/journal.pone.0056185 (PMC3575368; doi:10.1371/journal.pone.0056185)
Supplement: Methods S1 — Additional description of material and methods used or developed in this study. (DOCX) [file pone.0056185.s020.docx]

## SUPPLEMENTAL METHODS

**Exome Library Generation**

The sequencing libraries were prepared and captured using SureSelect Human All Exon 50Mb kit (Agilent Technologies) following the manufacturer’s instructions. Briefly, 3 µg of genomic DNA from each sample was fragmented by Adaptive Focused Acoustics (Covaris S2, Covaris, Inc., Woburn, MA) to produce an average size of ~175 bp. After repairing the ends, SOLiD sequencing adaptors (Life technologies) were ligated to the fragments and purified with Ampure Beads (Agencourt) for size selection and free adapter removal. In some cases the beads were left in the buffer after the elution step to maximize the recovery rate[1]. Each library was nick-translated, amplified by 6-cycles of PCR, and quantified by Bioanalyzer (Agilent). SureSelect solution hybridization was used to enrich the 50Mbp whole exome: 500 ng of each library was hybridized to the SureSelect baits during a 48 hrs. at 65ºC. The RNA-DNA duplex were then captured using MyOne Streptavidin beads (Life technologies) and the eluted material was further amplified using 8-cycles of PCR to add barcode tags.

**Sequencing**

Prior to sequencing, the libraries were quantified by qPCR using the SOLiD Library TaqMan Quantitation Kit (ABI) and the ABI 7900HT instrument (ABI). The barcoded samples were pooled in equimolar amounts in groups of six and the final pools were diluted to 500pM. The SOLiD EZ BeadTM system (ABI) was used to generate templated beads from the 500pM library pools. Approximately 900 million templated beads were deposited on a full slide. Sequencing was performed using the SOLiD 4 system, generating 50bp forward and 35bp reverse reads. One or two libraries were prepared for each sample, and sequenced across one to three runs, pooling together indexed libraries for the same patients on the same slide of the same run.

**Initial Data Analysis**

*Alignment:* All raw 50bp-35bp paired-end color-space reads were aligned to the human genome reference sequence (hg19), limited to chromosomes 1-22, X and Y, as well as mitochondrial genome. The alignment was carried out using BioScope v1.3.1 (Life Technologies Carlsbad, CA) with default parameters for paired-end reads and allowing for insertions and deletion within a read. We obtained more than 250M reads per sample (Table S1). Aligned reads were realigned using GATK’s IndelRealigner[2]. All reads from the same patient were combined and realigned together. The realigned reads were then split back into their respective samples and duplicate reads were removed using Picard Tools v1.53 MarkDuplicates. Picard misses some PCR duplicates due to the clipping of the ends of the reads by Bioscope; we detected these missed duplicates by identifying the true read-start/end coordinates using the CIGAR string of the aligned read. Reads with identical true start/end were considered duplicate and all but one were rejected. Finally the GATK’s TableRecalibration tool was used to recalibrate the reads’ base quality scores. *Coverage:* The coverage of the bases located on target, near target (+/- 250bp from target), or off target (>250 bp from target) bases was calculated using Picard Tools CalculateHsMetrics (Table S12). The normalized coverage was calculated by dividing the coverage at each base by the average exome-wide coverage for each sample (Figure S2).

**Identification of contaminating Mouse DNA in xenograft tumor samples**

In order to identify potential mouse DNA contamination of the xenograft tumor sample, the reads from xenograft samples were aligned to the mouse genome (UCSC mm9) using Bioscope with the same parameters used to align reads to the human genome (see above). We then compared the alignment to the human and mouse genome to remove reads belonging to the mouse genome (Figure S6). All reads can fall in the two following categories: 1) *Reads aligned only to one genome*: the reads aligned uniquely to the human genome only are kept. Reciprocally, the reads aligning uniquely to the mouse genome are discarded. 2) *Reads aligned to both genomes*: we looked at their respective matched (paired) read from the same fragment, which defined the following three sub-categories: 2a) the reads not properly paired in any genome are discarded; 2b) the reads properly paired in one genome only are kept (human) or discarded (mouse); 2c) the reads properly paired in both genome are then evaluated for their matching (M) score. The M score is calculated by summing up the number of perfectly matching bases (X) and subtracted the total number of mismatch bases (Y), M = X - Y. When comparing the read’s M scores between the human and mouse genomes, prioritizing, for each fragment, the 50bp read over the 35bp read. The read is kept (respectively discarded) if the 50bp read’s M score is higher in the human (respectively mouse) genome. In the case of an identical score from the 50bp read comparison, we apply the same criteria to the 35bp read. Reads from fragment with identical M scores on both reads in both genomes were kept.

**Calling Variants**

We used VarScan v 2.5.5 [3] to compare the tumor to the normal sample and identify, for each patient, the single nucleotide variants (SNVs) and small insertions and deletions (indels) that are inherited (germline variants), somatic mutations, resulting from a loss of heterozygosity (LOH) or of unknown status. We used the default parameters for filtering variants except changing the tumor, normal, and combined minimum coverage to 1X each (default values are 6, 8, and 8, respectively) and minimum average quality score to 0 (default 15). For all subsequent analysis on germline variants, we considered LOH variants as germline variants, since they are a specific class of heterozygous variants. By default, variants of unknown type are filtered out by VarScan. We restricted our analysis to the variants located in the targeted regions and implemented the following four successive filtering steps to improve their confidence.

*Filter 1: Filtering low quality indels:* we required a minimum coverage of 10X, a minimum of 3 reads supporting the indel, and a maximum of 5% of germline reads supporting the indel for somatic indels.

*Filter 2: Default VarScan filters:* we filtered out variants meeting any of the following criteria. 1) The variant is within 3bp of an indel, (false-positive variants caused by indels). 2) Three or more variants located within 10bp (false positives caused by missed indels or alignment errors). 3) A minimum alternate allele frequency of 20%. A lower alternate allele frequency decreased significantly the concordance with the genotyping array (data not shown).

*Filter 3: Remove variants with low average alternate allele base quality:* We removed false positive variants likely caused by sequencing errors as observed from the bimodal distribution of alternate allele base quality of the novel (not in dbSNP) germline SNPs (Figure S5). To remove variants with a low average alternate allele quality score, first we calculated the average alternate allele’s base quality score (AQ) at each SNV. Next a minimum AQ of a SNV was applied to germline, LOH, and somatic variants. To determine the minimum AQ score to use we clustered all SNV’s AQs using MCLUST assuming 2 distributions [4]. This resulted in two z scores (Z_1_ and Z_­2_) for each SNV. Z_1_ and Z_2_ are the probabilities of the SNV belonging to the first (low AQ) and second distribution (high AQ), respectively. We retained SNVs with Z_2_ ≥ 0.95.

*Filter 4: Remove low quality somatic variants:* To remove germline variants incorrectly called somatic, we requested a maximum of 5% alternate allele frequency in the normal DNA. Low confidence somatic variants were finally removed by applying a minimum right-tailed Fisher exact P-value of 0.05 as determined by VarScan.

After filtering we identified, in the targeted regions, an average of 27,934 germline variants and ~233 somatic mutations in the 8 tumor samples (Table S11).

**Somatic Variant Annotations**

SeattleSeq (http://snp.gs.washington.edu/SeattleSeqAnnotation/) was used to annotate somatic variants for function. To find relevant somatic variants, we compared our set of somatic variants to the COSMIC database version 55 [5]. We considered only mutations in the COSMIC database that met the following criteria: 1) the mutations were described in published literature (PubMed ID), 2) the mutations had valid genomic coordinates, and 3) the mutant allele was reported. We compared our set of discovered somatic mutations to the 151,502 entries of 4,542,623 total COSMIC entries that met these three criteria.

**Comparison of Primary Tumor and Tumor Model**

Similar to the comparison between germline and primary tumor samples to identify somatic variants (implemented in VarScan, see above), a two-sided Fisher exact test was used to determine if a somatic variant found in the primary tumor and/or the tumor model was unique to that sample or shared by both samples. We performed the test on the union of the somatic variants identified in the primary and model tumors for each patient. By comparing allele read support in each sample, the two-sided Fisher exact test was used to calculate the probability, P-value, that the variant is unique to a sample. To correct for multiple testing, the data was permuted 100 times by randomly shuffling the sample a read belongs to and using the randomly assigned reads to calculate a random P-value. Lastly, an FDR of 0.05 was used to filter the somatic variant’s based on their p-value. The resulting non-significantly different variants were called ‘shared constant’. The variants that were determined as significantly different were further split into ‘unique’ somatic variants (mutant allele frequency<5% in one sample) or ‘shared changing’ somatic variants (mutant allele frequency ≥5% in both samples).

**Estimation of the primary tumor purity**

In order to estimate the amount of normal DNA in the tumor DNA, we compared, for each patient, the mutant allele frequencies of somatic variants with ≥ 30X coverage that were shared between primary and model. Assuming that the model is 100% tumor, then, mAF_p_=(1/(1+c)) mAF_m_ where c is the fraction of normal DNA in the primary tumor and mAF_p_ and mAF_m_ are the mutant allele frequencies for the same somatic variant in the primary tumor and tumor model samples, respectively. We estimated c using a linear regression model between mAFp and mAFm assuming a null y-axis intercept. We estimated that SK01600, SK00115, SK00102 and SK00072 primary tumors have 9%, 11%, 25% and 41% purity.

**Identification of Copy Number Aberrations (CNAs)**

We used ExomeCNV version 1.2 [6] to call CNAs and LOH regions throughout the genome. The B allele frequencies inputted into ExomeCNV were on target germline heterozygous variants passing Filters 1-3. To improve the power of ExomeCNV’s CNAs calling algorithm, we used regions of DNA corresponding to bases located on target or near target (+/- 250bp). To minimize sequencing noise in the CNA calls caused by library or sequencing biases, we created a high confidence “normal” coverage by pooling all male normal samples (SK00115, SK00102, and SK00072) using ExomeCNV’s pool.coverage. CNAs were called by comparison to this normal coverage using the default parameters except for setting the contamination level (c) to 0.1, minimum coverage to 5X, and the read length (l) to 43. For calling LOH regions we used the “two.sample.fisher” method and the suggested parameters except for an alpha of 0.999 in each iteration of the CBS algorithm in the multi.LOH.analyze step. We then removed any copy number call (neutral, deleted, amplified) that did not meet the two following filters: 1) the CNA segment was < 100 Kb in length, 2) two targets within the CNA segment were > 10 Mb apart. This prevents spurious CNA calls due to the scattered and uneven distribution of the exome capture baits. Using this methodology, we noticed an excess of amplification in SK00115 neurospheres calls when compared to the matched germline. After close inspection, we observed that the sequencing of this sample led to a specific coverage bias, not found in the other samples. Specifically, the distribution of coverage depth as a function of the GC content of the probe showed a marked difference between SK00115 neurospheres and all other samples (Figure S3), although all experiments were performed in parallel. After correction by statistical regression, we were able to improve the calls (Methods). This observation indicates that calling CNAs using coverage information is highly sensitive to slight experimental variations despite the care taken to process matched samples identically.

*A chromosome arm* was called deleted or amplified if the sum of the size of the segments called deleted or amplified, respectively, represented ≥ 20% of the arm total length. To identify *high confidence focal CNAs* we first excluded CNAs segments present within a chromosome arm deletion or amplification. We then called high-confidence amplified (respectively deleted) the segment with a logR ratio higher (respectively lower) than the 95^th^ (respectively 5^th^) percentile of the logR ratio of copy neutral segments*. Cancer Gene Census Copy Numbers:* We extracted the copy number calls made by ExomeCNV for all 405 genes from the cancer gene census (02/12/2012 [7]) and removed any gene that was called copy number neutral in all 8 samples. For each of the 283 remaining genes, we calculated the average log R ratio as ${avg LRR}_{g}=\frac{\sum_{s=1}^{n} (\mathrm{LRR}_{s}*O_{g, s})}{\sum_{s=1}^{n} (O_{g, s})}$. Where s is the copy number segment outputted by ExomeCNV, LRR_s_ is the LRR for a given segment s, n is the total number of segments overlapping with gene g, and O (g,s) is the number of base pairs overlapping between segment s and gene g.

**References**

1. Fisher S, Barry A, Abreu J, Minie B, Delorey TM, et al. (2011) A scalable, fully automated process for construction of sequence-ready human exome targeted capture libraries. Genome biology 12: R1. doi:10.1186/gb-2011-12-1-r1

2. McKenna A, Hanna M, Banks E, Sivachenko A, Cibulskis K, et al. (n.d.) The Genome Analysis Toolkit: a MapReduce framework for analyzing next-generation DNA sequencing data. Genome Res 20: 1297–1303. doi:gr.107524.110 [pii] 10.1101/gr.107524.110

3. Koboldt DC, Chen K, Wylie T, Larson DE, McLellan MD, et al. (2009) VarScan: variant detection in massively parallel sequencing of individual and pooled samples. Bioinformatics 25: 2283–2285. doi:btp373 [pii] 10.1093/bioinformatics/btp373

4. Fraley C, Raftery A (2007) MCLUST Version 3 for R: Normal Mixture Modeling and Model-Based Clustering.

5. Forbes SA, Bhamra G, Bamford S, Dawson E, Kok C, et al. (2008) The Catalogue of Somatic Mutations in Cancer (COSMIC). Curr Protoc Hum Genet Chapter 10: Unit 10 11. doi:10.1002/0471142905.hg1011s57

6. Sathirapongsasuti JF, Lee H, Horst BAJ, Brunner G, Cochran AJ, et al. (2011) Exome Sequencing-Based Copy-Number Variation and Loss of Heterozygosity Detection: ExomeCNV. Bioinformatics (Oxford, England) 27: 2648–2654. doi:10.1093/bioinformatics/btr462

7. Futreal PA, Coin L, Marshall M, Down T, Hubbard T, et al. (2004) A census of human cancer genes. Nat Rev Cancer 4: 177–183.
